# The alcohol industry lobby and Hong Kong’s zero wine and beer tax policy

**DOI:** 10.1186/1471-2458-12-717

**Published:** 2012-08-30

**Authors:** Sungwon Yoon, Tai-Hing Lam

**Affiliations:** 1 Department of Community Medicine & School of Public Health, Li Ka Shing Faculty of Medicine, The University of Hong Kong, 21 Sassoon Road, Pokfulam, Hong Kong, SAR, China

**Keywords:** Alcohol tax, Alcohol industry, Public health policy, Hong Kong, Political tactics

## Abstract

**Background:**

Whereas taxation on alcohol is becoming an increasingly common practice in many countries as part of overall public health measures, the Hong Kong Special Administrative Region Government is bucking the trend and lowered its duties on wine and beer by 50 percent in 2007. In 2008, Hong Kong removed all duties on alcohol except for spirits. The aim of this paper is to examine the case of Hong Kong with its history of changes in alcohol taxation to explore the factors that have driven such an unprecedented policy evolution.

**Methods:**

The research is based on an analysis of primary documents. Searches of official government documents, alcohol-related industry materials and other media reports on alcohol taxation for the period from 2000 to 2008 were systematically carried out using key terms such as “alcohol tax” and “alcohol industry”. Relevant documents (97) were indexed by date and topic to undertake a chronological and thematic analysis using Nvivo8 software.

**Results:**

Our analysis demonstrates that whereas the city’s changing financial circumstances and the Hong Kong Special Administrative Region Government’s strong propensity towards economic liberalism had, in part, contributed to such dramatic transformation, the alcohol industry’s lobbying tactics and influence were clearly the main drivers of the policy decision. The alcohol industry’s lobbying tactics were two-fold. The first was to forge a coalition encompassing a range of catering and trade industries related to alcohol as well as industry-friendly lawmakers so that these like-minded actors could find common ground in pursuing changes to the taxation policy. The second was to deliberately promote a blend of ideas to garner support from the general public and to influence the perception of key policy makers.

**Conclusions:**

Our findings suggest that the success of aggressive industry lobbying coupled with the absence of robust public health advocacy was the main driving force behind the unparalleled abolition of wine and beer duties in Hong Kong. Strong public health alliance and advocacy movement are needed to counteract the industry’s continuing aggressive lobby and promotion of alcoholic beverages.

## Background

In September 2011, the United Nations High-Level Meeting on Non-Communicable Diseases highlighted harmful use of alcohol as one of the four major risk factors for non-communicable diseases [1]. Consistently ranked as one of the leading sources of disease burden, alcohol consumption is estimated to cause 2.5 million deaths each year globally [2]. Despite the alarming figures showing the negative health effects of alcohol, the global alcohol industry is continuing to rapidly expand into emerging markets with few or no alcohol control policies and regulations [3]. The industry has a huge capacity to market alcohol and promote drinking as part of an acceptable and “healthy” lifestyle. The potential for a rapidly growing alcohol epidemic is enormous given the increasingly ubiquitous availability of alcohol and aggressive promotion by the alcohol industry [4].

The World Health Organisation (WHO) suggests that alcohol taxation be a part of government efforts to regulate alcohol consumption. Studies have shown that the price of alcohol is significantly associated with consumption behaviour, particularly among heavy and underage drinkers [5]. Countries that raise the price of alcohol through taxation find that consumption correspondingly decreases. A large body of literature has also demonstrated that there is a strong inverse relationship between alcohol tax levels and alcohol-related harm and harmful drinking [6-9]. An analysis of the internal documents of the global alcohol industry confirms that alcohol taxes are one of the industry’s main concerns because tax increases lead to higher product prices and hence potentially reduce sales and profits [10,11]. Therefore, alcohol taxation is one of the most effective policy tools for controlling levels of alcohol consumption.

Whereas alcohol taxes have become common practice in many countries as part of public health measures, the Hong Kong Special Administrative Region (HKSAR) Government lowered its duties on wine and beer by 50 percent in 2007, and in 2008 removed all duties on alcohol except for spirits. Consequently, Hong Kong has become the only place in the world where there is neither a wine duty nor sales tax [12]. Recent announcements [13] by the alcohol industry indicate that they intend to seek tax exemption for spirits as well, while efforts to cultivate a “wine culture” and heavy alcohol advertising in Hong Kong indicate the massive growth envisaged by “market forces”. Today, by any measure, Hong Kong has relatively lower levels of alcohol consumption when compared with many other developed countries and regions. However, Hong Kong is seeing a rising trend in alcohol use, both in terms of the prevalence and drinking patterns. A recent government report shows that there was an increase of four percentage points in the prevalence of drinking among the adult population in Hong Kong from 30.9 percent in 2005 to 34.9 percent in 2010 [14]. The proportion of frequent binge drinkers (as reported drinking of at least 5 glasses/cans of alcohol beverages on one single occasion three times and more in the past month) increased from 35.0 percent in 2004 to 45.5 percent in 2011 [15,16].

The nature of public policy making in Hong Kong can be understood within a broad political context. As the novel concept of “one country-two systems” indicates, Hong Kong is a Special Administrative Region of China. The political arrangement in Hong Kong is defined by its constitutional document namely the *Basic Law of Hong Kong*, and the power to amend the Law lies with the National People’s Congress in Beijing [17]. Under the Basic Law, almost half of the Legislative Council members are derived through functional constituencies that represent the major business, professional, and social groups, and the other half are elected by universal suffrage. Hong Kong’s Chief Executive, the head of the Hong Kong Government, is elected by an Election Committee drawn mostly from the voters in the functional constituencies [18]. The constitutional constraints imposed by Beijing limit popular elections for the Legislative Council, thereby giving rise to the dominance of politically conservative business elites on the political scene. As such, this contributes to a situation where a small number of interest groups clustered around the functional constituencies have disproportionate sway over the HKSAR’s political scene. As we will see in this paper, these factors also contribute to the direction of public policy making with a strong emphasis on economic neoliberalism while at the same time marginalising the influence of the public’s interests.

The aim of this paper is to provide a chronological account of the changes in Hong Kong’s policy on alcohol taxation during the period from 2000 to 2008, with particular reference to the role of aggressive industry lobbying [19-21] as the main driving force behind the radical policy shifts (see Table 1). This paper begins with an account of major amendments to alcohol tax legislation in Hong Kong in 2000–2008. It then examines how the alcohol industry sought to strategically advance its vested interests to lower and eventually eliminate the beer and wine duty in Hong Kong. It discusses the nature of the alcohol industry lobby in terms of the employment of new political tactics as well as the propagation of a blend of ideas related to alcohol and alcohol consumption. The paper concludes by raising some questions concerning Hong Kong’s alcohol tax policy changes for future public health research and intervention.

**Table 1:** Chronology of events related to alcohol tax policy in Hong Kong (2000–2008)

**Year**	**Month**	**Event**
2000	June	A commissioned consultancy study on the assessment of Hong Kong’s potential to develop into a distribution and trading centre is completed by the Trade and Industry Bureau together with Hong Kong Trade Development Council (1999–2000).
2000	August	Financial Secretary Donald Tsang indicates Hong Kong is well placed to develop into a wine distribution hub for Asia.
2001	February	Financial Secretary Donald Tsang proposes to increase the duty rate on beer from 30 percent to 40 percent.
2002	February	Financial Secretary Anthony Leung proposes to increase the duty rate on wine from 60 percent to 80 percent.
2002		Coalition of alcohol industry actors (HKWSIC) begins to form.
2002	June	Legislator Tommy Cheung requests the government to provide a timeline for the development of wine hub
2004	December	The Financial Services and the Treasury Bureau launches a *Public Consultation on the Duty on Alcoholic Beverages.*
2006	January	The HKWSIC calls for alcohol tax cut in a media interview
2006	November	The HKWSIC urges the government to lower alcohol duties at press conference
2006	December	The Liberal Party and the Democratic Alliance for the Betterment and Progress of Hong Kong support the alcohol duty reduction
2007	February	The HKWSIC hosts a press conference to advocate the alcohol tax reduction
2007	February	Financial Secretary Henry Tang proposes to reduce the duty rate on beer from 40 percent to 20 percent, and on wine from 80 percent to 40 percent.
2008	January	The HKWSIC submits its proposal on Hong Kong as a wine hub to the Treasury Bureau
2008	February	The HKWSIC hosts a press conference to urge the government to slash alcohol taxes
2008	February	Financial Secretary John Tsang proposes to exempt the duties on wine and beer and all other alcoholic beverages except spirits.
2008	June	Amendment of Dutiable Commodities Ordinance Cap. 109 which provides for suspension of the licensing/permit requirement for import/export, storage, manufacturing and movement of the selected alcoholic liquors.

## Methods

The research for this paper was based on an analysis of primary documents. Official documents were retrieved from the Hong Kong Legislative Council and other government departments concerned for the period from 2000 to 2008. All government documents were available in Chinese and English bilingual versions. Key search terms such as “alcohol tax”, “wine hub”, “budget”, and “alcohol industry” were used and relevant threads contained in the documents were followed to search for additional documents of interest. The documents included budget speeches, annual statistics on alcohol consumption, consultation papers, customs data, press releases, population surveys, and transcriptions of Legislative Council meetings regarding the issue of alcohol taxation. These documents were reviewed to examine the chronological changes in the alcohol tax policy of the Hong Kong Government.

A literature search was conducted on a variety of alcohol-related industry websites, especially those based in Hong Kong. We began with broad search terms such as “alcohol tax”, and “wine hub”, and then extended our search to include additional terms identified in the documents reviewed. The industry documents offered insight into the alcohol industry’s tactics in its effort to abolish the alcohol tax as well as activities of key individuals and groups that worked closely with the industry. It should be noted that much of the industry information presented in this paper was obtained through a search of official industry documents comprising the alcohol industry’s submissions to various government departments, press releases, web-sites and industry bulletins. In this paper, the alcohol industry refers to multinational alcohol producers and wholesalers as well as retailers (off-sales) and hospitality sectors (on-sales) that sell alcohol products within Hong Kong. Media reports were obtained through the LexisNexis database for relevant coverage.

Initially, several hundred documents were found but the following categories of documents were regarded as ineligible: (1) excerpts of legislation and other government documents where alcohol duty rates are simply presented; (2) industry documents where the industry position is not mentioned; and (3) documents with information on other issues in Hong Kong. In total, 198 documents were retrieved. After excluding duplicates (several versions of one document, translations into English/Chinese, etc.), 97 documents most relevant to this study were examined in detail. These documents were further indexed by date and topic for a historical and thematic analysis using Nvivo8 software.

## Results

### The transformation of the alcohol tax regime

**Figure 1:**
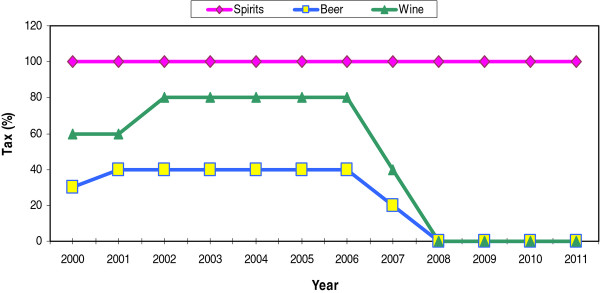
Alcohol Duty Rates in Hong Kong, 2000–2011.

In Hong Kong, alcohol duties have been applied on both imports and goods manufactured locally on an *ad valorem* basis but not levied on exports or re-exports. Throughout the 1990s, the duty rates on alcoholic beverages, except wines, remained unchanged: liquor with an alcoholic strength of more than 30 percent was taxed 100 percent while wine and beer were taxed at a rate of 60 percent and 30 percent, respectively. The alcohol taxation policies have, however, undergone dramatic transformations in the 2000s. In 2001, the Hong Kong government increased the duty rate on liquor with an alcoholic content of 30 percent and below (except wines) from 30 percent to 40 percent [22]. Subsequently, the government raised the duty on wine from 60 percent to 80 percent in 2002 (see Figure 1).

One of the main reasons for such a tax increase was the rise in Hong Kong’s budget shortfall, with a consolidated deficit of HK$ 65.6 billion (US$ 1 = HK$ 7.80) for 2001–02 as a result of the continuing slump in the property market and the local economy [22]. In view of the economic downturn, the excise tax represented a more politically viable way of raising revenue for the government than other taxes such as those on capital gains and income. In his 2001 budget presentation, then-Financial Secretary Donald Tsang (who was appointed Chief Secretary in 2001–2005 and subsequently assumed the office of Chief Executive in 2005–2012) acknowledged that he felt pressured because “Hong Kong must overcome the difficulties spawned by the economic downturn” [23]. As part of effective measures to restore the fiscal balance, he believed that an increase in the duty on beer would be inevitable. He stated that the tax increase would generate additional revenue of HK$ 90 million each year [23]. In the following year, faced with a growing budget shortfall, Financial Secretary Anthony Leung, Tsang’s successor, proposed to increase the duty rate on wine from 60 percent to 80 percent, which he claimed would produce an additional HK$ 70 million in revenue [22].

The increase in alcohol taxation was met with immediate opposition from the alcohol industry. From the alcohol industry’s perspective, the tax represented a direct menace to their continued prosperity. The Hong Kong Beer Industry Coalition (HKBIC), which then comprised seven international brewers and importers, strongly opposed the tax increase on beer. In May 2001, the HKBIC claimed that “such an increase would adversely affect the livelihood of several hundred thousand people working in the beer, retail and catering industries [24]”. More importantly, in order to gain wider public support, they highlighted that the tax increase might significantly push up retail prices, which would eventually penalize the majority of ordinary beer drinkers [25]. The HKBIC’s attempt to rally the general public against the tax increase was well reflected in their public statement: “The HKBIC does not believe that it is fair to impose a duty rate increase, which affects a broad spectrum of ordinary consumers, while the duty rates on luxury products remain unchanged [24].”

Likewise, the local wine industry fiercely opposed the duty increase and urged the government to reconsider its proposal. The Hong Kong Wine Industry Coalition (HKWIC), then comprising eleven wine manufacturers, employed a rather similar rhetoric to appeal to the middle class, as they could be construed as a potential supporter against government tax-rise legislation. In its letter submitted to the Legislative Council in April 2002, the HKWIC argued that “wine is a staple consumer good enjoyed by a wide range of the population” and therefore such an increase “affects a large cross-section of the population, not a small group of high-income earners [26].” They warned that the government would not receive the planned additional HK$ 70 million in revenue from the alcohol tax increase. Rather, they estimated that the tax increase would result in a loss of government revenue because there would be general signs of trading-down activities to cheaper priced alcohol [27]. Linking the wine duty with Hong Kong’s competitiveness vis-à-vis other economies in the region, the SoHo Association Limited representing the catering industry noted that raising the wine duty ran counter to encouraging investment in Hong Kong [28]. In a separate move, the Australian and New Zealand Consulate-Generals in Hong Kong, the two major suppliers of wine to Hong Kong, sent protest letters to the government. They argued that “consumers will react to the tax increase by switching to lower taxed and untaxed beverages and to illicit sources of smuggled wine, defeating the aim of substantially raising revenue [29].”

Despite the alcohol industry’s vigorous lobbying, the Hong Kong government was initially resistant to the industry’s opposition primarily because alcohol tax was seen as a “stable source of government revenue [23]”. On average, the alcohol tax had contributed approximately 0.4 to 0.5 percent of the government’s total revenue annually from early 2000 to 2005. For this reason, the tax rate on alcoholic beverages remained intact until the first half of the 2000s despite the industry’s political pressure and strong resistance.

### The corporate movement and new tax reduction strategies

By mid-2004, two factors appeared to have contributed towards a new policy climate that favored a reduction in the alcohol tax. One was the robust economic rebound despite the severe economic fallout caused in part by the outbreak of Severe Acute Respiratory Syndrome (SARS) in 2003. The Hong Kong economy exhibited a broad-based upturn in 2004 with a rise in real gross domestic product (GDP) of 8.7 percent amid a strong inflow of capital funds and an upsurge in consumer spending [30]. The other arguably more important factor was the persistent lobbying and political pressure from the alcohol industry. Over the years, the alcohol industry incessantly lobbied the government to lower the alcohol tax rate [31].

In its efforts to lower (and eventually abolish) the tax in question, the alcohol industry sought out industry allies such as the hospitality and trading industries and forged agreements with them to collectively advocate its position. Although forming alliances was not a new industry practice, it became an increasingly important strategy. Among others, the Hong Kong Wine & Spirits Industry Coalition (HKWSIC) was notable for this [32]. The coalition was first formed with the wine industry only and named as the Hong Kong Wine Industry Coalition (HKWIC) in 2002 “to lobby the government on industry related issues such as alcohol duties [33].” Then the alcohol tax increase in 2001–2002 prompted a more typical formation of like-minded industry groups which shared similar vested interests. Fredric Dufour, the managing director of Richemont Hong Kong, a transnational retailer of liquor products, tobacco and other luxury goods, became the first chairman of the HKWSIC [34].

In order to convince policy makers and political leaders, the coalition needed a representative that could provide legislative tax initiatives, help industry lobbyists gain access to lawmakers and senior government officials, demonstrate constituent support for alcohol tax reduction and testify on the industry’s behalf. Tommy Cheung Yu-Yan, who holds the seat of the catering industry functional constituency in the Legislative Council of Hong Kong, acted as a core policy link between the HKWSIC and the government, advocating for reductions in alcohol taxation.

At the Legislative Council meetings in 2002, he repeatedly delivered the message that the high cost of quality wines and spirits in Hong Kong damages Hong Kong’s tourism sector [35]. In order to convince politicians and government officials, the idea of Hong Kong becoming a regional hub in the alcohol trade was advanced. The coalition claimed that a lowered duty on wine would boost tourism and further strengthen Hong Kong’s image as a wine distribution centre [35]. The central idea was that a wine tax cut would encourage more investment from international wine traders, which would in turn result in more employment and economic growth.

Indeed, Hong Kong’s political leaders have long been interested in transforming Hong Kong into a regional wine trading centre [36]. In 2000, then-Financial Secretary Donald Tsang once mentioned that Hong Kong had great potential as a wine hub as the demand for wine in Asia and mainland China was rising [22]. The Hong Kong Trade Development Council estimated that the total market for wine in Asia was expected to grow at between 10 percent and 20 percent per annum to a probable consumption value of US$ 17 billion by 2012, and rising to US$ 27 billion by 2017 [37]. Such rosy estimates began to garner political support for building Hong Kong into a wine trading centre. For senior government officials who regarded rapid economic prosperity as the paramount political priority, this policy option was too attractive to resist. Through meetings with Legislative Council members as well as officials in the Financial Services and Treasury Bureau, the coalition provided a variety of supporting arguments and figures to substantiate their case [38].

Faced with relentless industry lobbying, the Hong Kong Government then began to consider assessing the existing alcohol duty regime. In December 2004, the government launched a *Public Consultation on the Duty on Alcoholic Beverages* to seek feedback on the appropriate level of alcohol taxation [39]. In its consultation document, the government indicated that while the government maintained its position that “the duty on alcoholic beverages should not be abolished but be retained as a form of tax,” a review of the alcohol taxation policy might be needed primarily because “there are constant calls from the liquor industry and the catering sector for a reduction in the duty on alcoholic beverages [40].” This statement clearly reflects the increasing pressure from the alcohol industry to lower the duty on alcohol.

Amidst signals from the government that alcohol tax reduction was under consideration as a likely option, the alcohol industry had high hopes, leading it to establish task forces, mobilize more public campaigns, and assemble arguments [41]. The coalition urged the government to lower the tax on alcohol by at least half. The campaign had gathered momentum after signs of a government revenue surplus appeared from 2005 onwards. The increase in government revenue was a politically excellent opportunity for the industry to raise the issue of lowering alcohol duties once again. The coalition noted, in January 2006, that “this is about the time to push forward our proposals as the economy is recovering steadily. We hope the government can reduce the duties when the public has more money to spend [42].”

Since late 2006, the industry elected to work closely with local media in an attempt to garner the wide spectrum of public support. For instance, daily newspaper titles such as “80 percent tax on wine too high, says lawmaker” or “hospitality industry says it would pass on saving to long-suffering consumers” were indicative of how the coalition sought to garner and increase the level of public support [43,44]. The need for a reduction in the alcohol tax was often illustrated in a simple form of fact sheets, press releases and Q & A materials in order to move the public sentiment on alcohol taxes [45]. Using industry data, the media often highlighted the relatively high alcohol prices in Hong Kong compared to neighboring regions such as Mainland China and Macau, so as to shape the public perception that the alcohol tax was an unfair form of taxation [46]. In December 2006, Fat-Long Chan, co-chairman of HKWSIC, asserted in the media that “Hong Kong has the highest alcohol duties in Asia. Many consumers decide to purchase alcohol abroad and the government loses taxes. If alcohol duties are decreased, we will also decrease our alcohol prices [47].”

The coalition’s opposition to alcohol duties was based on the protection of their commercial interests, but arguing that such duties hurt sales was not a viable political strategy. Instead, the industry attempted to build public support for its position by defining the alcohol duties as a threat to employment and the local economy. A local newspaper cited legislator Tommy Cheung’s remarks that the high alcohol tax threatened to harm employment: “[should the existing tax regime continue], the wine business will have to cut its profit margins to be competitive. It may result in unnecessary lay-offs and future [economic] difficulties [43].”

Boris de Vroomen, the new co-chairman of the HKWSIC and managing director of Moet Hennessy Diageo Hong Kong, was keen to gain a broader spectrum of support by allying with a range of actors as well as communicating with the public. In a media interview in December 2006, he stated that:

"*We are trying to engage with other parties as well to increase the level of support. Demonstrating that this [alcohol duty reduction will lead to reduction in alcohol prices] is good for the general public in Hong Kong can lead to a majority of Legco [Legislative Council] supporting this, and then I think the financial secretary will be on board as well *[44]*.*"

The Liberal Party and the Democratic Alliance for the Betterment and Progress of Hong Kong supported the move [48]. Sixteen consul-generals had sent a joint letter to the financial secretary backing the call for lower alcohol duties [44]. On 6 February 2007, shortly before the Financial Secretary’s 2007–2008 budget speech, the HKWSIC held a media briefing on the need for lowering alcohol taxes. Tommy Cheung was presented as an influential leader to advocate for the coalition’s position. Cheung argued that “we strongly want the government to reduce duties on wine. We have been talking about this for years. Once the city becomes a wine centre, everyone will come to buy [49].”

On the other end of the spectrum, a small number of lawmakers and commentators expressed concerns about the negative effects of alcohol tax reduction. In January 2006, at a Legislative Council meeting, the Democratic legislator Fred Lo Wah-Ming presented his concerns about possible drinking problems among young people should the existing tax regime change [42]. In September 2006, amidst the growing climate of favouring a reduction in taxes on alcohol, Vladmir Poznyak, the WHO coordinator for management of substance abuse, suggested that Hong Kong use taxation on alcoholic beverages as part of an alcohol control policy. He further noted that the beneficial effects of moderate drinking had been over-rated: “we should be cautious about the message that red wine is good for the heart [50].” Writing in a local newspaper in December 2006, Professor Hildemar Santos, a public health physician at the Tsuen Wan Adventists Hospital, stated that growing availability of alcoholic beverages through reduction in retail prices may increase alcohol-related harms. Comparing to the effects of tobacco taxation on smoking, he further asserted that:

"*My argument is based on the fact that most countries that increased tobacco taxes saw a bigger decrease in smoking than with any other public health anti-smoking campaign. I believe the converse is true for all potentially addictive substances: lower taxes lead to higher consumption. It is therefore unwise to push for lower taxes on anything that is addictive and abusable *[51]*.*"

Despite a few other similar calls for the institution of alcohol taxation to play a bigger role as part of public health and alcohol control measures, the message has not been effectively communicated.

### Towards a zero tax regime in the absence of public health advocacy

In 2007, Hong Kong saw a remarkable budget surplus of more than US$ 7 billion with a 6.8 percent GDP growth rate. In his budget speech on 28 February 2007 [52], then Financial Secretary Henry Tang announced that taxation on all forms of alcohol except spirits would be lowered by 50 percent with immediate effect, costing the government HK$ 350 million per year. This meant that the duty on beer and other types of liquor containing no more than 30 percent alcohol was reduced to 20 percent from the previous 40 percent, and the wine duty was adjusted to 40 percent from the previous 80 percent. Tang believed that “reducing the duty will help promote the development of our catering industry, tourism and wholesale and retail alcoholic beverage trade, thereby benefiting the community at large [52].” He further stated that he was willing to “consider the innovative idea of abolishing alcohol duty to boost economic activities and promote development of Hong Kong as the regions’ wine exhibition trade and logistics centre [52].”

Whereas halving the duty on wine and beer was welcomed warmly by the industry [53,54], there were little, if any, concerns raised from public health experts and civil society organizations (CSOs). In recent years, local non-governmental organizations (NGOs) have played an increasingly important role in exerting pressure on relevant government policies and gaining public support. In the field of public health and environment, NGOs have been, in cooperation with academics and others, progressively active in various aspects of the policy-making scene. For instance, independent non-profit organizations such as the *Hong Kong Council on Smoking and Health*, the *Clear the Air*, and the *Civic Exchange* have been active and committed to tobacco control and/or environmental protection as part of their advocacy [55]. They engaged stakeholders and the wider community in campaigns to protect and promote public health in Hong Kong. Their efforts have resulted in several positive outcomes such as the expansion of smoke free places in January 2007 under the revised Smoking (Public Health) Ordinance, raising public awareness of Hong Kong’s poor air quality, and increasing the tobacco duty by 50 percent in 2009 and 41.5 percent in 2011 [56].

In view of the roles that local NGO actors actively played in the making of public health policy, it is surprising that they have apparently been totally absent in the policy debate on alcohol taxation. The lack of civil participation in creating policy dialogues and raising concerns related to alcohol tax reduction lies in stark contrast to the vociferous mobilization and lobbies driven by the industry. Apart from concerns raised by a professor of public health about the impact of alcohol tax reduction on drinking behaviour amongst young people, the social and public health issues surrounding alcohol taxation were given little attention. Sian Griffiths, Director of the School of Public Health of the Chinese University of Hong Kong, warned in a media interview with a local newspaper in March 2007 that a low alcohol tax might translate into a higher availability of alcoholic drinks, which in turn would potentially increase alcohol consumption, particularly among younger drinkers who are more price-sensitive [57].

In reviewing the industry materials, it is apparent that the coalition devoted particular attention to the positive health effects of wine drinking. Massive publicity and aggressive alcohol promotion efforts were based upon the notion that consuming a small amount of alcohol, in particular wine, helps protect against diseases of the heart [58]. Regardless of the scientific validity and the controversy of the health benefits of drinking wine, this notion has substantially created the widespread misconception among the public that wine is less associated with alcoholism and by implication allegedly less harmful than beer or spirits. How the notion of the positive health effects of red wine was widely accepted among the local public can be found if one looks at the consumption patterns of Hong Kong drinkers. In 2007, retained imports of red wine amounted to US$ 102.9 million and that of white wine were US$ 13.7 million, representing a ratio of 7.5 to 1 [59]. The Hong Kong-based wine industry’s document published in March 2007 is also indicative of Hong Kong drinkers’ perception on the beneficial cardiovascular effects of red wine consumption: “Drinking about two glasses of wine a day is beneficial to health and that is a major influence on the boom of the wine market in Hong Kong…Hong Kong drinkers prefer red wine to white wine because of more perceived health benefits associated with drinking red wine [60].”

On the policy front, the pro-business Liberal Party has been a key force behind the idea that drinking wine is healthy. At the heart of this move stood Party Chairman James Tien Pei-Chun. Shortly after the alcohol tax reduction, he called for further tax cuts on wine. At the Legislative Council meeting held on 28 March 2007, he claimed that one of the reasons why the wine tax should be eliminated was that unlike other alcoholic drinks, wine is beneficial to one’s health. Tien argued that:

"*Many medical professionals and doctors are of the view that the consumption of red wine is different from that of whisky or brandy, for the alcoholic content of red wine is relatively low and its effect on our liver and kidney is smaller. Unlike smoking, the consumption of red wine is not hazardous to our health *[61]*.*"

In collaboration with business-friendly lawmakers, the alcohol industry employed a variety of instruments including reports, conferences, meetings, opinion pieces and letters to lucidly articulate the rationale for alcohol tax elimination [62]. In its proposal submitted to the Treasury Bureau in January 2008, the coalition claimed that abolishing the wine duty would generate HK$ 4 billion a year in sales and related businesses such as wine storage and fine wine actions [63]. The coalition held a press conference on 2 February 2008, where they urged the government to scrap the wine duty and halve taxes on spirits to 50 percent [64]. Tommy Cheung said that abolishing the alcohol tax would “drive Hong Kong to be a regional wine trading centre and bring economic benefit to Hong Kong [64].” The extensively publicized health benefits of moderate wine consumption had served to underscore their argument.

The corporative initiative appeared to have convinced the government officials and senior policy makers. On 27 February 2008, Hong Kong entered a new era as it implemented a zero alcohol taxation policy. In his budget speech, newly appointed Financial Secretary John Tsang announced that he would scrap all duties on wine and beer. This move has made Hong Kong the only place in the world where wine and beer are completely untaxed [65]. Tsang stated that the aim of the action was to attract more commercial opportunities and investments to Hong Kong in the alcohol trade, costing the government HK$ 560 million annually in lost revenues [66]. Effective 6 June 2008, a new regulation that eased permit controls was implemented under the amended Dutiable Commodities Ordinance [67]. This policy removed the need to obtain licenses and permits to sell liquor with less than 30 percent alcoholic strength and wine, thus creating a more conducive commercial environment.

The HKWSIC commended the decision, claiming that “a bottle of wine will now be cheaper in Hong Kong than anywhere else in Asia. It will make Hong Kong into a sensible hub for exporting, primarily into China [68].” The economic deregulation and liberalization of licensing, alongside the industry’s publicity to cultivate the wine culture, is likely to induce a change towards a wine drinking culture. One US alcohol industry document clearly indicates that its publicity efforts would target the changing drinking practices in Hong Kong. It states that “Hong Kong drinkers are getting more and more receptive to wine drinking practice. The total elimination of the excise tax on wine would probably help nurture wine drinking culture in Hong Kong…. It is an excellent opportunity for US wine traders to expand their exports [69].” As anticipated, wine consumption among the adult population in Hong Kong has dramatically increased in terms of the volume of pure alcohol consumed from 1,588,901 litres in 2004 to 3,164,107 litres in 2008 [14].

Intriguingly, following the reduction (and elimination) of wine and beer duties, alcohol consumption per capita in Hong Kong has slightly increased from 2.54 litres in 2006 to 2.64 litres in 2010 [14]. In particular, a surge in alcohol consumption per capita (3.00 litres) was observed in 2008 when duties on wine and beer were totally abolished. Since the implementation of a zero wine and beer tax policy, there have been calls for the Hong Kong government to lower the duty on distilled spirits by changing the current duty system [70]. The government did not accept the proposal on the grounds that the current system is simpler and fairer and is in line with the ability to pay principle. The government stated that any reform to the current system should avoid any regressive effects and other possible unfair situations between products of different price ranges [71]. Nonetheless, the HKWSIC, which represents the distilled spirits industry in Hong Kong, continues to pursue further tax reform that will effectively lower the duty.

## Discussion

This paper examined the role of the alcohol industry in the changes to alcohol tax policy in Hong Kong. More specifically, we investigated how the industry actors sought to successfully influence policymaking in an effort to abolish the duties on alcohol. The conversion of Hong Kong from an environment of high alcohol taxation to a duty-free zone was done with remarkably high speed and efficiency over a two year period. Although the city’s changing financial circumstances and the Hong Kong Government’s strong propensity towards economic liberalism have in part contributed to such a dramatic transformation, the alcohol industry’s tactics and strategies were clearly the main drivers of the policy decision. Whereas the alcohol industry had been constantly vociferous in lowering the alcohol tax, the second half of the 2000s witnessed striking changes in the political tactics and rhetoric that they employed to lobby for selective amendments to licensing laws and to the ways in which alcoholic products were taxed.

To comprehend how the alcohol industry successfully advanced their vested interests and carefully positioned themselves in the policy-making arena, it is important to identify the political tactics that they employed. From the experience of Hong Kong, the industry’s political tactics were two-fold. The first tactic was to forge a new coalition. The alcohol industry lobbying as a whole was not tightly integrated and coherent right from the beginning. The industry actors came to realize that individualized and haphazard lobbying techniques were not effective in shaping the views of legislators or the public on alcohol tax issues. The alcohol industry, notably the HKWSIC, employed a tactic that was designed to encompass a range of alcohol-related catering and trade industries so that these like-minded actors could form common ground in the pursuit of changing the taxation policy.

Over the years, coordinated efforts among these actors were fundamental to facilitating action, representing their shared interests about how alcohol tax policy should be changed, and determining the outcome of policy debates towards a zero-alcohol tax. The formulation of alcohol tax policy was effectively facilitated by the bridges the coalition built with politicians to allow for a broader and more coordinated array of influence. Our findings clearly point to a prominent role played by a few business-friendly legislators such as Tommy Cheung and James Tien on the policy-making scene. They acted as what could be known as “policy brokers”, whose positions and bargaining power enabled them to influence outcomes of policy debates to their advantage [72,73]. The very fact that these policy elites represent business interests speaks of the homogeneity of the coalition. By managing coordinated interactions of constituent actors and acting in a concerted manner, the coalition was able to achieve such unprecedented success.

The second tactic was to deliberately employ the communication programme and publicity that might fit into the overall aim of making the city a tax-free environment. The coalition needed to develop an appropriate blend of ideas to lobby for the abolition of taxes on alcohol. While the underlying policy goal of the alcohol industry was to foster corporate preferences, calling for tax reductions publicly as such was not ideal. Therefore, a message was carefully presented in such a way that alcohol tax reduction would benefit Hong Kong as a whole. The coalition’s message was structured around four key ideas which were closely tied in with the coalition’s corporate interests.

The first idea was that tax reduction would stimulate Hong Kong’s economy, boost tourism and thus enhance employment prospects. More specifically, the coalition maintained that Hong Kong should be the region’s centre for wine exhibition, trading and logistics. The core argument was that removing wine duties would encourage more investment from international wine traders, which would in turn create more jobs and bring other economic benefits to Hong Kong. Second, in an attempt to gain public support, it was claimed that excessive alcohol duties deprived ordinary Hong Kong drinkers of the enjoyment of affordable alcoholic beverages. Newspaper interviews and other speaking opportunities were strategically used to carefully shape the public perception that a cut in alcohol taxation would vastly benefit consumers through a reduction in retail prices. The third idea advanced by the industry coalition was that Hong Kong’s high alcohol tax drives consumers to lower jurisdictions to buy alcoholic beverages, thereby losing revenue for the government and reinforcing the illegal smuggling and illicit activities connected with the alcohol market. Indeed, the alcohol industry repeatedly propagated an idea that should the existing tax be maintained, the government would not achieve its intended increase in revenue as a result of alcohol smuggling and trading-down effects toward the consumption of cheaper alcohol. Fourth, moderate consumption of wine was framed as conducive to good health. Although the beneficial effects to the heart of moderate consumption of red wine are contentious, the alcohol industry communicated these benefits to the public in a very aggressive manner.

Our findings reveal that policymakers appeared to have been swayed by the industry’s argument, notably the economic merits of their proposition. The resultant outcome was that the alcohol tax policy in Hong Kong was interpreted and formulated solely in the trade arena rather than in the domain of public health. Because government officials and policy elites saw alcohol as an ordinary commodity [74], it was commonly believed that eliminating taxes on alcohol and expanding for commerce would be beneficial to the city as a whole. In particular, the idea of turning Hong Kong into the fine wine hub for the Asian market sharply attracted government authorities to the extent that policies for tax reduction and elimination could be executed over such a short period of time. The new licensing act clearly reflected the government’s inclination towards a market-based approach in view of what is seen as the booming demand for wine from Asia and mainland China [75].

Regrettably, where the alcohol tax policy was framed and justified strictly in fiscal/economic terms, an alternative argument from CSOs was barely present. Because the rhetoric of competitiveness, free markets, and economic prosperity dominated political and policy agendas, there was little room for CSOs to counteract commercial interests. Such general lack of counter arguments on the tax reduction from the community at large appeared to facilitate the implementation of the zero-tax policy. Additionally in Hong Kong, CSO activities have traditionally been confined to certain public health issues such as tobacco control and air pollution. Defining alcohol as a public health concern emerged only recently after the zero alcohol tax was implemented [14,76]. Hong Kong’s experience in alcohol tax policy changes sheds light on a scenario where in the absence of strong civil and public health advocacy, the alcohol industry assumes the key role in policy making [77]. Put bluntly, the real and potential harms to public health by alcohol misuse are being insidiously cast aside by the industry players in their bid to pursue vested interests with the misleading claims of health benefits.

From the perspective of public health, it is important to debunk the alcohol industry’s misleading narratives, and to critically assess the impact of the Hong Kong Government’s policy decision on the zero beer and wine tax. In this sense, this paper raises more questions than it answers. For instance, is the overall trend in early-age alcohol uptake and alcohol consumption by the youth in Hong Kong expected to escalate since they are more sensitive to a drop in price? What would be the external costs of the abolition of the alcohol tax? Can we observe any changes in crime, homicide, domestic violence, suicide, and productivity losses associated with the consumption of alcohol? More fundamentally, even if the abolition of the tax on alcohol would bring economic benefits, what would be the gains and losses in government revenue and what are the opportunity costs foregone? What would be the long-term public health implications and resultant social costs of such a policy? In terms of the provision of public goods, would the economic benefits of the abolition of the alcohol tax adequately compensate for this? How are the economic benefits distributed in Hong Kong society to justify such a sacrifice in terms of public health? Would the profits earned be concentrated in the hands of a few companies that only profess verbally to have Hong Kong’s interests at heart? From a regional perspective, to what extent does the transformation of Hong Kong into a wine hub affect alcohol use in neighboring areas in Asia and Mainland China? Does the regional public health community follow the impact and prepare other countries to anticipate similar industry initiatives since Hong Kong provides an excellent industry playbook? Hong Kong is a unique opportunity to examine such effects as no other jurisdiction has adopted such an unprecedented policy of alcohol taxation.

## Conclusions

This paper explored the successful influence of the alcohol industry in shaping the policy agenda and determining the policy outcome towards a tax-free environment for alcohol in Hong Kong. It reviewed key tactics and rhetoric employed by the alcohol industry to lobby for the abolishment of alcohol duties. This paper illustrates that in the absence of public health advocacy, the alcohol industry is able to successfully form alliances, frame policy issues on alcohol, and directly exert influence on policy making. This suggests that strong public health alliances and advocacy are needed to counteract the industry’s aggressive lobbying and promotion and to demonstrate the spuriousness of the industry’s claims [78]. The present case study from Hong Kong indicates the urgency for research and actions on alcohol control.

## Abbreviations

HKBIC: The Hong Kong Beer Industry Coalition; HKSAR: Hong Kong Special Administrative Region; HKWIC: The Hong Kong Wine Industry Coalition; HKWSIC: The Hong Kong Wine & Spirits Industry Coalition; CSO: Civil Society Organizations; NGO: Non-Governmental Organizations; WHO: World Health Organisation.

## Competing interests

The authors declare that they have no competing interests.

## Authors’ contributions

SY performed the analysis and drafted the manuscript. THL conceived of the study and revised the manuscript. All authors read and approved the final manuscript.

## Pre-publication history

The pre-publication history for this paper can be accessed here:

http://www.biomedcentral.com/1471-2458/12/717/prepub
